# *O*^6^-(4-bromothenyl)guanine reverses temozolomide resistance in human breast tumour MCF-7 cells and xenografts

**DOI:** 10.1038/sj.bjc.6602833

**Published:** 2005-11-08

**Authors:** M Clemons, J Kelly, A J Watson, A Howell, R S McElhinney, T B H McMurry, G P Margison

**Affiliations:** 1 Cancer Research UK Carcinogenesis Group, Paterson Institute for Cancer Research, Wilmslow Road, Manchester M20 9BX, UK; 2 Cancer Research UK Department of Medical Oncology, Christie Hospital NHS Trust, Wilmslow Road, Manchester M20 4BX, UK; 3 University Chemical Laboratory, Trinity College, University of Dublin, Dublin 2, Ireland

**Keywords:** MGMT, temozolomide, breast cancer, MCF-7, breast tumour xenografts

## Abstract

Tumour resistance to chemotherapy involving methylating agents such as DTIC (dacarbazine) and temozolomide is linked to expression of the DNA repair protein *O*^6^-alkylguanine-DNA alkyltransferase (MGMT). There is considerable interest in improving the efficacy of such *O*^6^-alkylating chemotherapy by the prior inactivation of MGMT. We have examined the effect of the modified guanine base, *O*^6^-(4-bromothenyl)guanine (PaTrin-2, Patrin™, Lomeguatrib) on MGMT activity and cell or xenograft tumour growth inhibition by temozolomide in the human breast carcinosarcoma cell line, MCF-7. PaTrin-2 effectively inactivated MGMT in MCF-7 cells (IC_50_ ∼6 nM) and in xenografts there was complete inactivation of MGMT within 2 h of dosing (20 mg kg^−1^ i.p.) and only slight recovery by 24 h. MGMT inactivation in a range of murine host tissues varied between complete and ∼60%, with extensive recovery by 24 h. PaTrin-2 (10 *μ*M) substantially increased the growth inhibitory effects of temozolomide in MCF-7 cells (*D*_60_=10 *μ*M with PaTrin-2 *vs* 400 *μ*M without). In MCF-7 xenografts, neither temozolomide (100 mg kg^−1^ day^−1^ for 5 days) nor PaTrin-2 (20 mg kg^−1^ day^−1^ for 5 days) had any significant effect on tumour growth. In contrast, the PaTrin-2–temozolomide combination produced a substantial tumour growth delay: median tumour quintupling time was increase by 22 days (*P*<0.005) without any significant increase in toxicity as assessed from animal weight. A PaTrin-2–temozolomide combination may therefore be beneficial in the treatment of human breast cancers.

Standard first-line chemotherapy for advanced breast cancer results in disease regression in the majority of patients, but these responses are rarely complete or sustained (Clemons *et al*, 1997). There is, therefore, a continuing need to develop new treatments and one potential strategy is the biochemical modulation of tumour drug resistance.

The chemotherapeutic methylating (e.g. dimethyltriazenoimidazole-4-carboxamide (DTIC)) and chloroethylating (e.g. bischloroethylnitrosourea (BCNU)) agents are well established in oncology and are a component of high-dose therapy regimens used in the treatment of breast cancer (Peters *et al*, 1994; Antman, 2001; Bengala *et al*, 2001). These agents are cytotoxic principally by virtue of their ability to alkylate DNA at the *O*^6^ position of guanine, and there is now considerable evidence that the DNA repair protein *O*^6^-alkylguanine-DNA alkyltransferase (MGMT) plays a key role in determining tumour resistance to these drugs (Margison and Santibáñez Koref, 2002; Gerson, 2002, 2004). MGMT repairs alkylation at the *O*^6^ position on guanine by accepting the alkyl group onto a cysteine residue in its active site in a stoichiometric and autoinactivating reaction. Cellular resistance *in vitro* and *in vivo* is correlated with elevated MGMT expression, and can be achieved in previously susceptible cell lines and organisms by transfer and expression of cDNAs or genes encoding MGMT. Furthermore, inactivation of MGMT, or gene deletion in mice, renders resistant cells and tissues sensitive to *O*^6^-alkylating agents (Pegg, 1990; Margison *et al*, 2002, 2003; Margison and Santibáñez Koref, 2002; Gerson, 2002, 2004).

Attempts to exploit this clinically have used methylating agents to deplete MGMT, via the formation of *O*^6^-methylguanine (*O*^6^-meG) in DNA, prior to the administration of a chloroethylating drug (e.g. Clemons *et al*, 2003). These have been hampered by the similar toxicities of the two agents, and no useful increase in therapeutic index has been demonstrated (Micetich *et al*, 1992; Lee *et al*, 1993; Smith *et al*, 1996; Hammond *et al*, 2004). Because of this, interest has turned to inactivation of MGMT using inherently nontoxic pseudosubstrates for the protein, such as *O*^6^-benzylguanine (*O*^6^-BeG; reviewed in Pegg *et al*, 1995; Dolan and Pegg, 1997) and *O*^6^-(4-bromothenyl)guanine (PaTrin-2, Lomeguatrib; McElhinney *et al*, 1998; Middleton *et al*, 2002; Middleton and Margison, 2003).

Temozolomide has recently been tested in breast cancer patients using a 5-day regimen and was found to be ineffective (NCIC, 2001). In order to establish if this resistance can be reversed in model systems, we have used the human breast adenocarcinoma cell line, MCF-7, that, like many of the breast tumours we have examined, expresses very high levels of MGMT. We show that PaTrin-2 inactivates MGMT in cells and xenografts and that this results in substantial increases in their sensitivity to growth inhibition by temozolomide.

## MATERIALS AND METHODS

### Drugs

PaTrin-2 and temozolomide were provided by the Cancer Research Campaign Drug Formulation Unit, University of Strathclyde, Glasgow, UK. For cell culture studies, a stock solution of PaTrin-2 (20 mM) was prepared in dimethylsulphoxide (DMSO; Sigma, Poole, Dorset, UK) and diluted into culture medium just prior to use. For animal studies, PaTrin-2 was ground to a fine powder and suspended in corn oil at 4 mg ml^−1^ immediately prior to intra-peritoneal (i.p.) injection. Temozolomide was freshly prepared at 40 mg ml^−1^ in 20% DMSO/80% phosphate-buffered saline (PBS) and diluted in 0.9% NaCl solution.

### Cell culture studies

MCF-7 cells (a human breast adenocarcinoma cell line) were grown as a monolayer in RPMI medium containing 10% foetal bovine serum (Gibco BRL, Paisley, Scotland), at 37°C in a humidified atmosphere of 5% CO_2_/95% air.

To determine MGMT inactivation, cells (5 × 10^6^) were incubated in the presence of increasing concentrations of PaTrin-2 at 37°C, 5% CO_2_. After 2 h, cells were pelleted and resuspended in 10 ml PBS. This was repeated three times in order to remove any residual PaTrin-2. Finally, cells were pelleted and assayed for MGMT activity as previously described (Watson and Margison, 2000). Activity remaining, based on at least three points on the linear part of the protein-dependence curve, was calculated as a percentage of the activity in untreated cells.

To determine toxicity, the MTT [3′ (4,5-dimethylthiazol-2-yl)-2,5-diphenyl tetrazolium bromide] growth inhibition assay, based on the method of Carmichael *et al* (1987) was employed. Cells (1000 per well) were plated into a 96-well plate and following a 24 h attachment period, PaTrin-2 was added to the cells. After 2 h incubation with PaTrin-2 (10 *μ*M) at 37°C, 5% CO_2_, increasing doses of temozolomide or vehicle were added and the cells were incubated for a further 4–5 days. At the end of the exposure period, 150 *μ*g MTT was added to each well and plates were incubated for 3 h at 37°C, 5% CO_2_. The media were removed and the formazan crystals formed in the viable cells were solubilised in 200 *μ*l DMSO. The absorbances at 540 and 690 nm were determined using a Titertek Multiscan ELISA plate reader and growth inhibition calculated as a percentage of the *A*_540_–*A*_690_ of untreated wells.

### Animal studies

Male nude mice (O/Nu: outbred ALPK Nu/Nu) were purchased from Zeneca (Macclesfield, UK). Animals were housed in a sterile environment and allowed free access to food and water. MCF-7 human breast tumour xenograft samples (1–2 mm^3^) were implanted in the right flank while the mice were under ethrane and halothane anaesthesia and the experiments begun when tumour volumes had reached a suitable size (see below). As MCF-7 tumours are oestrogen receptor positive, they required additional oestrogen for growth. To prepare oestrogen pellets, *β*-oestradiol (468 mg) was added to 9.7 g silastic and mixed. Curing agent (1.1 g) was added and the mixture spread into three (26 mm × 12 mm × 1 mm) glass formers. These were then incubated at 42°C overnight before being cut into 2 mm × 2 mm × 1 mm cubes (∼2 mg oestradiol per pellet). The pellets were stored at 4°C until insertion subcutaneously at the tail base, simultaneously with the tumour implant and monthly thereafter. Agents were injected i.p. within 15 min of preparation: Temozolomide was injected 1 h after PaTrin-2 or the vehicle control. Animals were cared for in accordance with Home Office guidelines

To measure MGMT depletion following PaTrin-2, six groups of mice with at least five mice in each group received PaTrin-2 20 mg kg^−1^ i.p. as a single dose. At varying times after dosing, animals were terminated by cervical dislocation, and the tissues (xenograft, kidneys, liver and lungs) dissected out and immediately frozen in dry ice. Bone marrow was collected from femora, which were dissected from each mouse. A minimum amount of bone was trimmed from each end and a 21-gauge needle inserted through the epiphyseal cartilage and the bone marrow flushed into an appropriate volume of PBS. Tissue was stored at −70°C until assayed for MGMT activity.

To assess the ability of PaTrin-2 to sensitise human breast tumour xenografts to the tumour growth inhibitory effects of temozolomide, groups of at least six nude mice were treated as follows: the vehicle control group were given corn oil then 20% DMSO in PBS; the temozolomide only group were given corn oil then temozolomide (100 mg kg^−1^ day^−1^); the PaTrin-2 only group were given PaTrin-2 (20 mg kg^−1^ day^−1^) then DMSO in PBS, and the PaTrin-2 plus temozolomide group were given PaTrin-2 (20 mg kg^−1^ day^−1^) then temozolomide (100 mg kg^−1^ day^−1^). Drugs or vehicles were administered i.p. once daily for 5 days with a separation of 1 h. Up to 10 and at least six animals were assigned to each group, and mean tumour volume was standardised across the groups at the start of the experiment: thus the control, PaTrin-2, temozolomide and PaTrin-2 plus temozolomide groups had mean tumour volumes of 29.8±7.6 (range 19.0–38.7), 33.2±14.7 (range 16.5–58.7), 35.1±10.9 (range 20.9–52.4) and 30.3±10.0 (range 20.7–44.5) mm^3^, respectively.

In all the tumour growth delay experiments, animal weights and tumour volumes were measured twice per week. Tumour volumes were calculated using the formula (length × height × width × Π/6) with measurements taken using digital calipers.

The relative tumour volumes were plotted for each animal and tumour quintupling times (tqt) determined. The statistical significance of differences between treatment groups was evaluated using the Mann–Whitney test. The maximum relative weight losses observed in each group were compared using the Kruskal–Wallis test.

## RESULTS

### Effect of PaTrin-2 on MGMT activity and temozolomide sensitivity in MCF-7 cells

**Figure 1:**
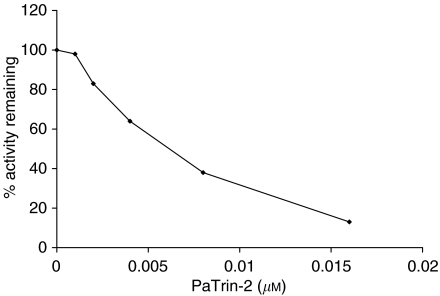
Effect of PaTrin-2 on MGMT activity in MCF7 cells. Values shown are determined from the slope of the activity assay curves and are effectively the mean of at least triplicate estimations. See text for experimental details.

MCF-7 cells expressed high levels of MGMT (∼1540 fmoles mg^−1^ total protein). Exposure to PaTrin-2 for 2 h resulted in extensive inactivation of MGMT in MCF-7 cells: the concentration required to inactivate 50% of the MGMT was around 6 nM (Figure 1).

**Figure 2:**
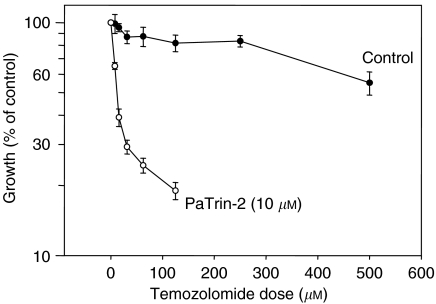
Effect of PaTrin-2 (10 *μ*M) on the sensitivity of MCF7 cells to the growth inhibitory effects of temozolomide. See text for experimental details.

The sensitivity of the MCF-7 cells to the growth inhibitory effects of temozolomide was substantially increased by PaTrin-2. Growth amounting to 60% of control was seen after 400 *μ*M temozolomide alone but following preincubation with 10 *μ*M PaTrin-2, 60% growth occurred at 10 *μ*M temozolomide (Figure 2), indicating a 40-fold increase in sensitivity. PaTrin-2 itself had no growth inhibitory effect.

### Effect of PaTrin-2 on MGMT activity in host tissues and tumour

**Figure 3:**
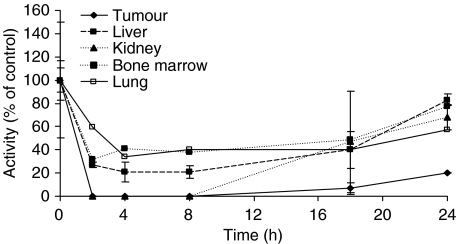
Kinetics of MGMT depletion in MCF-7 xenografts and other tissues after a single intraperitoneal dose of PaTrin-2 (20 mg kg^−1^). Points are the means of values (±error (s.d.) for tumour, liver, and kidney) from at least five mice. In order to have sufficient material for assay, bone marrow was pooled from five mice (no s.d. is shown). See text for experimental details.

Extensive depletion of MGMT activity was seen in all host tissues measured after a single i.p. dose of 20 mg kg^−1^ PaTrin-2 (Figure 3). Depletion to below the limits of detection occurred in the kidney, while it was to ∼20, ∼35 and ∼40% of pretreatment values in liver, lung and bone marrow, respectively. The nadir was generally between 2 and 8 h and substantial activity (to over 50% of pretreatment levels) had returned by 24 h after dosing. In the MCF-7 xenografts, complete MGMT inactivation was seen between 2 and 8 h, and recovery of levels was only to ∼20% of pretreatment levels by 24 h after dosing. Slower recovery in the xenograft might reflect the relative strength of the human MGMT promoter or that the human protein is more extensively inactivated by PaTrin-2 or its putative metabolites.

### Effect of PaTrin-2 and temozolomide on MCF-7 tumour growth

**Figure 4:**
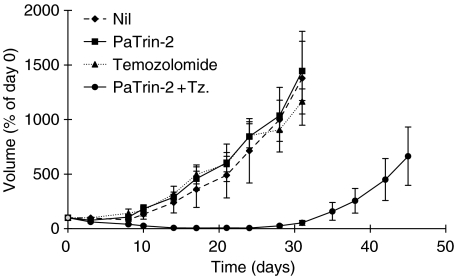
Growth of MCF-7 tumour xenografts in nude mice. Treatment was once daily for 5 days. Points are the means (±s.e.m.) of values from at least five mice. See text for experimental details. Tz=temozolomide.

**Figure 5:**
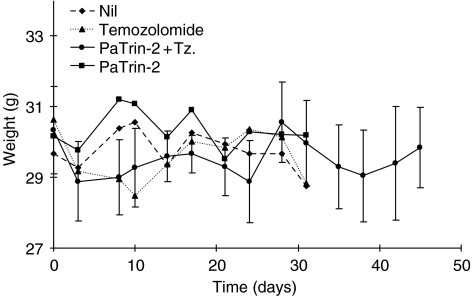
Weight change in nude mice receiving different temozolomide/inactivator combinations. Points are the means from at least five mice. Error (standard deviation) bars are shown only for the PaTrin-2 plus temozolomide group: these are representative of all other groups for which they have been omitted for clarity. Tz=temozolomide.

The median MCF-7 tqt in the vehicle control and PaTrin-2 only groups were ∼21 and ∼17 days, respectively. Neither temozolomide (tqt∼17 days) nor PaTrin-2 alone had any significant effect on xenograft growth. However, the combination of PaTrin-2 and temozolomide resulted in a median tumour quintupling time of ∼43 days representing an increase of ∼22 days (*P*<0.005; Figure 4, Table 1). Toxicity, as measured by weight loss, was essentially unaffected by the addition of PaTrin-2 to the temozolomide treatment regimen. At the end of the treatment period, weight loss was ∼5% in both the temozolomide alone and combination groups (Figure 5, Table 1).

## DISCUSSION

The *O*^6^-alkylating agent, temozolomide has been approved for the treatment of malignant glioma and is under consideration for use in melanoma, for which it is extensively prescribed, off license (e.g. Kiebert *et al*, 2003). It has also been tested in patients with metastatic breast cancer (NCIC, 2001) and prostate cancer (van Brussel *et al*, 2000) but inherent drug resistance has resulted in no clinical benefit. Several groups have measured MGMT expression in human breast tumours and report activity that is low (Cao *et al*, 1991), moderate (Chen *et al*, 1992) or high (Musarrat *et al*, 1995). High levels of MGMT expression in the primary tumour have been correlated with a poor prognosis in early breast cancer, irrespective of the treatment regimen (Citron *et al*, 1994). We have previously reported almost uniformly high levels of MGMT in breast tumours as determined by functional assay and immunohistochemical analysis (Clemons *et al*, 2002). It seems reasonable to suggest that resistance to temozolomde was mediated by these high levels of expression of MGMT.

While the *O*^6^-alkylating agents are currently not as widely used in the management of breast cancer as they are in glioma, malignant melanoma and lymphoma, the possibility of overcoming alkylating agent resistance would make these agents useful additions to the chemotherapeutic management of this much more common malignancy. Attempts to improve the effectiveness of *O*^6^-alkylating agents therapy by prior inactivation of MGMT using methylating agents have foundered owing to the similar, and hence cumulative, toxicities of the inactivating and treatment drugs (Lee *et al*, 1993; Smith *et al*, 1996; Clemons *et al*, 2002; Hammond *et al*, 2004).

Strategies have therefore focussed on the use of nontoxic MGMT inactivators and it has been shown that *O*^6^-BeG and PaTrin-2, which very effectively inactivate MGMT in cells and tumours, improve the therapeutic effect of alkylating agents in a number of tumour models (Dolan and Pegg, 1997; Wedge *et al*, 1997; Middleton *et al*, 2002). Phase I clinical trials of *O*^6^-BeG in combination with BCNU have shown increased myelosuppression, requiring reduced doses for phase II studies (Spiro *et al*, 1999; Schilsky *et al*, 2000; Friedman *et al*, 2000). Indeed, Chinnasamy *et al* (1997) found that *O*^6^-BeG pretreatment of mice significantly increased the *in vivo* cytotoxicity and clastogenic effects of BCNU in bone marrow. It has also been shown that the granulocyte–macrophage precursor cells of primary human bone marrow samples are dramatically sensitised to the toxic effects of temozolomide by pre-exposure to *O*^6^-BeG (Fairbairn *et al*, 1995; Clemons *et al*, 2000). So far, phase II clinical trials have yet to demonstrate that *O*^6^-BeG provides an increase in therapeutic index, that is, that the improvement in efficacy outweighs any additional toxicity (Quinn *et al*, 2002).

PaTrin-2 is a more potent MGMT inactivator *in vitro* than *O*^6^-BeG, and we considered it worthwhile to examine the extent to which PaTrin-2 could inactivate MGMT and increase sensitivity to temozolomide in a human breast tumour model as a prerequisite for any potential clinical trial in breast cancer. Our results show that PaTrin-2 is also a potent inactivator of MGMT in MCF-7 cells both in culture and in xenografts *in vivo.* Tumour MGMT depletion by PaTrin-2 was as extensive as was reported with *O*^6^-BeG in other tumour types (see Dolan and Pegg, 1997). We also showed that MGMT was inactivated in all host tissues with complete inactivation in kidney and extensive inactivation in other tissues. This collateral depletion again raises the concern about the potentiation of toxicity in healthy tissues following PaTrin-2/alkylating agent combinations.

The MGMT inactivation by PaTrin-2 in MCF-7 cells resulted in marked sensitisation to temozolomide growth inhibition. Following implantation into immune deficient mice, the resulting xenografts are completely resistant to growth inhibition by a 5-day treatment regimen using temozolomide alone. This was probably a result of the resistance conferred by high levels of expression of MGMT. PaTrin-2 alone had, as anticipated, no effect on tumour growth rates. However, PaTrin-2 overcame the resistance to temozolomide producing highly significant tumour growth delays, but without increasing toxicity as judged by animal weights. Thus, the therapeutic index of temozolomide is increased by PaTrin-2 in this animal model. We have previously shown that human melanoma xenografts expressing moderate levels of MGMT do respond to growth inhibition by temozolomide, but this is considerably enhanced by pretreatment with PaTrin-2 (Middleton *et al*, 2000, 2002). Current phase I studies are investigating the dose of PaTrin-2 that is necessary for complete inactivation of MGMT in patients with a variety of cancer types, prior to phase II studies.

Given the results of the xenograft studies with melanoma, and now breast cancer, it seems reasonable to speculate that the greatest benefit from PaTrin-2-mediated inactivation of MGMT might be seen in tumours with the highest levels of MGMT expression and inherent resistance to temozolomide. This might best be assessed in a phase II clinical trial in breast cancer, particularly since temozolomide alone has been shown to be ineffective in the MCIC trial. However, given the variable levels of expression of MGMT in this tumour type, it would be beneficial to assess MGMT levels in tumour biopsies from all patients in such a study so that the hypothesis that MGMT inactivation will be beneficial can be effectively tested.

## Figures and Tables

**Table 1:** Effect of temozolomide±PaTrin-2 on human breast MCF-7 xenograft growth and animal weight

**Treatment group**	**Growth delay (days)^a^**	**Weight losszz at end of treatment (%)**
Vehicles	0	1.2
PaTrin-2	−3	ND
Temozolomide	−3.3	4.7
Both drugs	29.4^b^^,^^c^	4.8

a Tumour growth delay is the difference between the median time for tumours in treated or control animals to quintuple in size (tumour quintupling time: tqt in text) relative to the vehicle control.

b *P*=0.005 compared to PaTrin-2 alone by Mann–Whitney test.

c *P*=0.003 compared to temozolomide alone by Mann–Whitney test.
